# Ocrelizumab-Induced Severe Colitis

**DOI:** 10.1155/2020/8858378

**Published:** 2020-12-07

**Authors:** Hsing Hwa Lee, Naveen Sritharan, Daniel Bermingham, Gabriela Strey

**Affiliations:** ^1^Department of Medicine, Hervey Bay Hospital, Pialba, Queensland, Australia; ^2^Department of Pharmacy, Hervey Bay Hospital, Pialba, Queensland, Australia

## Abstract

We report a case of severe ocrelizumab (anti-CD20 monoclonal antibody) induced colitis in a 43-year-old woman with multiple sclerosis leading to total colectomy after failing medical therapy. Histology of the colectomy was consistent with medication-induced colitis.

## 1. Introduction

Ocrelizumab is a humanized monoclonal antibody that binds to the CD20 antigen on B lymphocytes and has been approved for the treatment of primary progressive multiple sclerosis (PPMS) and relapsing multiple sclerosis (RMS) in adults [1]. Ocrelizumab-induced colitis has been reported to occur within a few weeks of initiation of therapy. In this case, severe colitis involving a significant portion of the colon and requiring total colectomy occurred after only two doses of ocrelizumab.

## 2. Case Report/Case Presentation

A 43-year-old Caucasian female presented with multiple episodes of loose, watery, dark stools. Her presentation was associated with gradual onset, colicky, lower abdominal pain since the previous day. She otherwise denied any fever, nausea, or vomiting, history of ill contact, or any history of travelling or taking outside food. No one within her household had similar symptoms.

The patient had been diagnosed several years earlier with multiple sclerosis (MS) and is wheel-chair-bound due to that disease. She had been prescribed alemtuzumab in a clinical trial 10 years preceding this presentation and had used teriflunomide for one year prior to being prescribed ocrelizumab by her neurologist—she had received two doses six months apart. She denied any prior history of inflammatory bowel disease or bowel cancer.

Upon examination, it was noted that she was afebrile and was haemodynamically stable. Examination of the abdomen revealed a soft abdomen with tenderness at the suprapubic and iliac regions with increased bowel sounds but no evidence of peritonism or organomegaly. Digital per rectal examination established an empty rectum.

**Figure 1:**
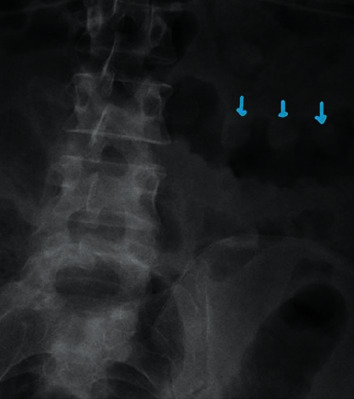
Abdomen X-ray showing thumbprinting sign.

The patient was ordered a range of initial laboratory and imaging investigations and follow-up diagnostics based on those findings (Tables 1 and 2). The abdominal X-ray (Figure 1) demonstrated a classical thumbprinting sign that was nonspecific but suspicious for clostridium difficile infection. Empirical antibiotics IV metronidazole and oral vancomycin were commenced for presumed clostridium difficile infection while awaiting for laboratory tests and histology results.

**Figure 2:**
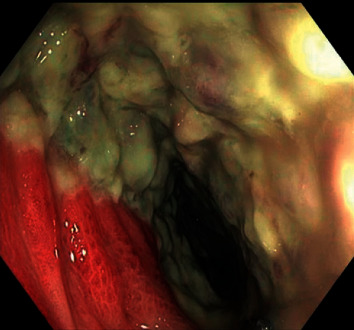
Distal sigmoid colon showing severe colitis.

**Figure 3:**
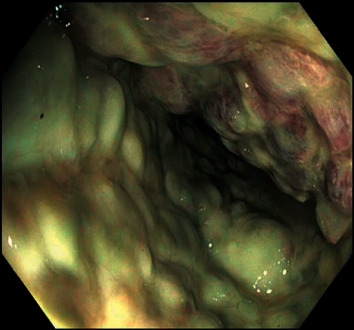
Midsigmoid colon showing extension of the severe colitis to the midsigmoid region.

The patient's venous blood gas showed lactate of 4 mmol/L (normal range, 0.6–1.8 mmol/L) which prompted a CT abdomen. The CT demonstrated a pronounced colitis extending from the transverse colon to the sigmoid colon. When the patient did not respond to IV metronidazole and oral vancomycin, a flexible sigmoidoscopy was undertaken and showed nodular mucosa with white-yellowish adherent plaques with increasing severity from the rectosigmoid to the sigmoid (Figures 2 and 3). The histology from biopsies taken from various parts of the left colon was negative for cytomegalovirus (CMV) and clostridium difficile infection, but suggestive of biological medication effect.

Accordingly, the patient was treated as ocrelizumab-induced colitis and commenced on intravenous hydrocortisone. Her CRP was downtrending on hydrocortisone. However, serial abdominal X-rays demonstrated ongoing gaseous distension, and she continued having watery bowel motions. The patient was referred to a tertiary hospital and colorectal surgeons for medication-resistant biological medication-induced colitis. She subsequently underwent a total colectomy and ileostomy formation.

## 3. Discussion/Conclusion

We surmise that the most likely cause of colitis in this instance is ocrelizumab, as evidenced by both the clinical history and histopathological reports. Drug-induced colitis is a relatively new clinically recognized phenomenon with diagnosis increasing in alignment to the expansion of medications and novel therapy. However, to date, we are aware of two other cases of de novo colitis associated with ocrelizumab, with one of them requiring surgical resection [2, 3].

Ocrelizumab is a humanized monoclonal antibody that binds to the CD20 antigen on B-lymphocytes and has been approved for the treatment of primary progressive multiple sclerosis (PPMS) and relapsing multiple sclerosis (RMS) in adults [1]. Ocrelizumab-induced colitis has been reported to occur within a few weeks of initiation of therapy. In this case, severe colitis involving a significant portion of the colon and requiring total colectomy occurred after only two doses of ocrelizumab. This may indicate either a cumulative effect from ocrelizumab or a delayed presentation of worsening colitis from commencement of the medication. Rituximab, which is also a humanized anti-CD20 monoclonal antibody, has been reported to be associated with colitis. However, fulminant colitis is extremely rare [4].

Ocrelizumab is likely to play a role in the development of autoimmunity resulting in severe pancolitis as described in our case. The exact mechanism of anti-CD20-induced colitis is not known, nor is the pathogenesis of ocrelizumab-induced colitis. Since ocrelizumab depletes B cells, there could be an association between colitis with the dysregulation of the gastrointestinal immune system via B-cell depletion. B cells play a vital role in the mucosal immune system by producing secretory antibodies IgA and IgM which protects the mucosal barrier [5]. B-cells also produce IL-10 which is an anti-inflammatory and has been linked to inhibit Th1-derived responses. Animal studies have shown that mice with depleted B-cells and IL-10 have severe colitis likely mediated by Th1 response profile [6]. Ocrelizumab-suppressed B-cell levels have been shown to return to pretreatment levels at a median of 72 (27–175) weeks [7]. Therefore, the inflammatory process with T-cell dysfunction may occur and persist for up to a year. Ocrelizumab could potentially predispose patients to develop autoimmunity by causing immune dysregulation. This process might continue in the background until another immunological trigger or disruption which leads to the development of autoimmune process.

Risk factors for ocrelizumab-induced colitis remain unclear. Several cumulative risk factors may have exponentiated the presentation in this particular case. This patient had previously received teriflunomide and alemtuzumab which may have altered her immune system which concomitantly could have been affected by an already proinflammatory state of multiple sclerosis. This is consistent with literature [2, 3] describing other cases where patients received ocrelizumab after failing first-line therapy. Whether this increased risk is due to a severe refractory disease state or simply the alteration of the immune system from multiple agents remains unknown.

Treatment of ocrelizumab-induced colitis is challenging as this is extremely rare, and hence, therapeutic options are limited. Immunosuppressants such glucocorticoids were only effective in one case report but not effective in two others, including our case, and surgical intervention was needed for definitive management as a life-saving procedure [2, 3]. More studies need to be done to explore other medical therapies such as other immunosuppresants or immunomodulators which could be another option. It is unclear what the best therapeutic options are due to the novelty of this condition. However, medical management and the least invasive management will likely produce the best outcome.

In conclusion, colitis may be a potential risk with administration of ocrelizumab. Research is required to identify patients at risk and devise strategies to monitor patients who could potentially develop complications from ocrelizumab.

## Figures and Tables

**Table 1:** Laboratory results.

Laboratory tests	Patient's sample	Normal values
Haemoglobin	172	115–160 g/L
White blood cells	15.2	4.00–11.0 × 10^9^/L
Platelets	160	140–400 × 10^9^/L
C-reactive protein (CRP)	187	<5.0 mg/L
Lipase	25	<60 U/L
Albumin	40	35–80 g/L
Lactate	4.0	0.6–1.8 mmol/L
Faecal calprotectin	3200	<50 *µ*g/g
Stool culture	Normal colonic bacterial flora	
Clostridium difficile toxins	Negative	

**Table 2:** Imaging and procedural results.

Imaging and procedures	Relevant findings
X-ray abdomen	Dilated large bowels with thumbprinting sign
CT abdomen	Pronounced colitis primarily involving the transverse colon, splenic flexure, and descending colon of the large bowel extending to involve the sigmoid colon
Flexible sigmoidoscopy	Decreased mucosa vascular pattern in the sigmoid colon
	Nodular mucosa in the rectosigmoid colon and in the sigmoid colon
	Congested, erythematous, inflamed, and vascular pattern decreased mucosa in the rectum. Histology showed features of biological medication effect.
Laparoscopy converted to open total colectomy	Widespread inflammatory adhesions with contact bleeding. Attempted laparoscopic resection converted to open total colectomy due to disintegrating fulminant colitis of left colon and sigmoid colon falling to pieces on retraction, contact bleeding, and massive distension of large bowel. Frank pus from rectum during insertion of rectal catheter at the end of the case.
Histology of total colectomy	Mucosa in proximal colon appears normal. The rest of the colonic mucosa is completely ulcerated without residual islands of intact mucosa. There is congestion, chronic inflammation, and submucosal fibrosis. The ulceration is mostly superficial but does extend focally to the muscularis propria. Focal areas of subserosal fibrosis are noted. The pathology features are compatible with the clinical diagnosis of medication induced colitis.
